# Genetic Diversity of *IGHM* and *IGHE* in the Leporids Revealed Different Patterns of Diversity in the Two European Rabbit Subspecies (*O. cuniculus algirus* and *O. c. cuniculus*)

**DOI:** 10.3390/ani9110955

**Published:** 2019-11-12

**Authors:** Ana Pinheiro, Tereza Almeida, Pedro J. Esteves

**Affiliations:** 1 CIBIO Centro de Investigação em Biodiversidade e Recursos Genéticos, InBio Laboratório Associado, Universidade do Porto, Campus Agrário de Vairão, 4485-661 Vairão, Portugal; tjvalmeida@gmail.com (T.A.); pjesteves@cibio.up.pt (P.J.E.); 2 Departamento de Biologia, Faculdade de Ciências, Universidade do Porto, 4169-007 Porto, Portugal; 3 CESPU, Instituto de Investigação e Formação Avançada em Ciências e Tecnologias da Saúde, 4585-116 Gandra PRD, Portugal

**Keywords:** European Rabbit, IgM, IgE, leporids, genetic diversity, immunoglobulins constant region

## Abstract

**Simple Summary:**

The study of European rabbit immunoglobulin genes has contributed decisively to the current knowledge on antibody structure and diversification. The European rabbit has also been increasingly used as an animal model for the study of many human diseases, such as syphilis, tuberculosis, and AIDS. As such, the study of its immune system genes is of crucial relevance, but the study of rabbit immunoglobulins has focused only on the IgG and IgA antibodies. In this study, we added to the knowledge of the rabbit immune system by investigating the genetic diversity of two antibodies, IgM and IgE, in wild and domestic rabbits as well as other rabbit close species. With the data obtained in this study, we showed a high similarity between the different rabbit close species studied and we pointed out important genetic differences in the wild and domestic rabbits. Our findings are a valuable tool for the management of rabbit wild populations and domestic breeds and may contribute to the identification of immunoglobulins genetic variants with greater efficiency against pathogens.

**Abstract:**

The European rabbit (*Oryctolagus cuniculus*) has been an important model for immunological studies but the study of its immunoglobulins (Ig) has been restricted to its unique IgA and IgG. Here, we studied the genetic diversity of IgM and IgE in several species of leporids and performed population genetics studies on European rabbit wild populations and domestic breeds. The leporids sequencing showed that these Ig are well conserved (98% sequence similarity among leporids), For IgM the Cµ1 and Cµ4 were the most diverse and most conserved domains, respectively, while for IgE the Cε1 was the most diverse domain and Cε2 and Cε3 the most conserved domains. The differences in the pattern of most conserved and most diverse domain between the Ig isotypes are most likely related to each isotype function. The genetic population data showed contrasting results for IgM and IgE. For both Ig, as expected, a greater diversity was observed in the original species range, the Iberian Peninsula. However, unexpectedly the genetic diversity found for IgE in the domestic animals is higher than that for the French wild populations. These results will increase knowledge of the genetic diversity of leporids and wild and domestic rabbit populations and are important tools for the management of wild populations and rabbitries.

## 1. Introduction

Immunoglobulins (Igs) are one of the most emblematic components of the jawed vertebrate immune system. These heterodimeric glycoproteins act as a critical part of the immune system by recognizing and binding to antigens and subsequently triggering mechanisms that lead to its destruction [1]. In eutherian mammals, five Ig isotypes have been described, IgM, IgD, IgE, IgG and IgA. Differences between Ig isotypes reside in the constant domains of the heavy chain (CH) and determine that each isotype has specific effector functions [1]. 

**Figure 1:**
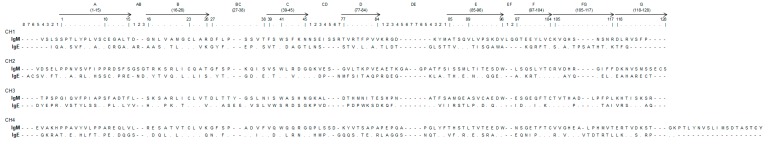
European rabbit (*Oryctolagus cuniculus*) IgM and IgE DNA sequences of constant regions (Genbank accession numbers for IGHM: J00666, and IGHE: AY386696.1). The amino acid translation is shown. The IMGT unique numbering for the constant domain (13) is shown on top.

The European rabbit (*Oryctolagus cuniculus*) has been used as an animal model for diseases and immunological studies (reviewed in [2]). One of the peculiarities of the European rabbit is the unique features of Ig isotypes. As some other mammals the European rabbit has no IgD and no functional *IGHD* gene has been found in the rabbit germline [3,4]. IgM and IgE are encoded by single genes, *IGHM* and *IGHE*, as well as IgG, a surprising fact among mammals that typically have multiple *IGHG* copies encoding multiple IgG subclasses [4,5]. For IgA, on the other hand, the European rabbit has at least 15 *IGHA* genes encoding 15 IgA subclasses [6,7] being the most complex IgA system of all studied mammals. These unique aspects may, perhaps, explain that the study of European rabbit Igs has focused on IgG and IgA (e.g., [6,7,8,9,10,11]) forgetting other Ig isotypes. In fact, the information available for European rabbit IgM and IgE is extremely scarce with only a few sequences available in public databases (Figure 1).

IgM, in its transmembrane form, is the first isotype to be expressed during B-cell development. As such the VH and VL regions associated with this Ig have not undergone much antigenic driven diversification being able to recognize a wider range of antigens than other isotypes, although showing low affinity [1]. In mammals, IgM is secreted in pentamers, in which the IgM monomers are linked by disulfide bonds in the CH4 domain and further bound a J chain. This pentameric form is very effective in opsonizing pathogens and IgM antibodies are also very efficient at fixing complement [12]. Immunoglobulin E (IgE) has the lowest serum concentration and shortest half-life of all Ig subclasses. Despite this, it is a very potent immunoglobulin associated with hypersensitivity, allergic reactions and response to parasitic worm infections [1]. 

The European rabbit originated on the Iberian Peninsula (IP) [13], where two subspecies coexist: *O. cuniculus algirus* in the south-west area of the IP and *O. cuniculus cuniculus* in the north east area. After the last glacial peak, *O. c. cuniculus* expanded its range north, across the Pyrennes, towards France [14], from where the species was domesticated in the last 1500 years [15,16]. It belongs to the family Leporidae which comprises 11 genera: *Brachylagus, Bunolagus, Caprolagus, Lepus, Nesolagus, Oryctolagus, Pentalagus, Poelagus, Pronolagus, Romerolagus* and *Sylvilagus*) [17,18]. Most are monotypic genera with restricted distributions, with the exception of *Lepus*, a polytypic and cosmopolitan genus, and *Nesolagus*, *Pronolagus* and *Sylvilagus*, which comprise two, four and more than 16 species, respectively [17,19,20,21,22,23,24,25]. Despite some discrepancies in leporid taxonomy are still unresolved, the widest consensus agrees that the Leporidae first basal group included *Pronolagus*, and that *Romerolagus* is at the second basal position [26,27,28]. 

In this study, we extend the knowledge on IgM and IgE isotypes in leporids by sequencing the complete *IGHM and IGHE* genes for six additional extant leporid genera: *Bunolagus*, *Brachylagus*, *Lepus*, *Pentalagus*, *Romerolagus* and *Sylvilagus*. Additionally, we compared the genetic diversity of these Igs between wild rabbit populations of *O. c. algirus* and wild and domestic populations of *O. c. cuniculus.*

## 2. Material and Methods

**Figure 2:**
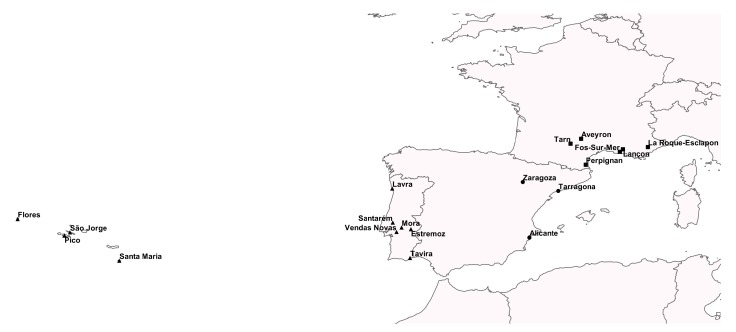
Map with the geographical locations of the populations used in this study. Marked with (●) are *O. c. algirus* populations from Portugal, with (■) are *O. c. cuniculus* from Spain and with (▴) *O. c. cuniculus* from France.

For the study of *IGHE* and *IGHM* evolution in leporids, we analysed specimens of *Bunolagus, Brachylagus, Lepus, Pentalagus, Romerolagus* and *Sylvilagus* genera. To assess the genetic diversity of European rabbit we analysed 10 individuals for each of three wild populations: *O. c. algirus* collected in Portugal and *O. c. cuniculus* collected in Spain and in France (Figure 2); as well as 15 domestic animals from 6 breeds: French Lop, Flemish Giant, Argent Champagne, Angora, New Zealand White and Netherland Dwarf. Total genomic DNA was extracted from frozen liver or ear tissue using an EasySpin Genomic DNA Minipreps Tissue Kit (Citomed). No animals were killed for this study. All samples used in this work belong to the Cibio/InBio sample collection and had been previously used in other works [11,29,30].

PCR amplifications of the four *IGHE* and *IGHM* exons was conducted using primers designed on the basis of European rabbit available sequences (GenBank accession number AY386696) [4]. A fragment containing the IGHE CH1 and CH2 domains was amplified using primers FE12 5’AGGTGGAAGCCAGGGTGAGG 3’ and RE21 5’ CGCCTCGCGGTTCGTAATCT 3’ under the following conditions: 15 min at 95 °C followed by 35 cycles at 95 °C (30 s), 60 °C (30 s) and 72 °C (45 s), with a final extension at 60 °C (20 min). Another fragment containing IGHE CH3 and CH4 exons was amplified using primers FE31 5’ TGACACCCAGAAAGACAGGG 3’ and RE41 5’ CCGAGTGACACTGCAGTGTT 3’ under the following conditions: 15 min at 95 °C followed by 35 cycles at 95 °C (30 s), 62 °C (30 s) and 72 °C (45 s), with a final extension at 60 °C (20 min). A fragment containing the IGHM CH1 and CH2 domains was amplified using primers FM11 5’ AGCTTTTCACACCTCCCCTT 3’ and RM21 5’ AAACCCATGAGGACGCCTGT 3’ under the following conditions: 15 min at 95 °C followed by 35 cycles at 95 °C (30 s), 62 °C (30 s) and 72 °C (45 s), with a final extension at 60 °C (20 min). Another fragment containing IGHM CH3 and CH4 exons was amplified using primers FM31 5’ TCTGGGTGAAACCACCCCTT 3’ and RM41 5’ CTGACAGGGTTAGTTTGCAT 3’ under the following conditions: 15 min at 95 °C followed by 35 cycles at 95 °C (30 s), 58 °C (30 s) and 72 °C (45 s), with a final extension at 60 °C (20 min). Sequences were determined by automated sequencing following the Big Dye Terminator Cycle Sequencing protocol (Perkin Elmer, Warrington, UK) using the referred primers. 

Sequences obtained in this study were edited and aligned using CLUSTAL W [31] as implemented in BioEdit software [32] and the amino acid sequences were inferred using the software BioEdit [32]. The obtained sequences were also aligned and compared to European rabbit sequences available in GenBank. Codon numbering is according to the IMGT unique numbering for C-DOMAIN [33].

Sequence nucleotide diversity was estimated using DnaSP version 5.10 [34]. Haplotype reconstruction was obtained for each individual using PHASE version 2.1 [35] as implemented in DnaSP version 5.10 [34]. DnaSP was used to estimate population genetic parameters (S, π), Tajima’s D, number of haplotypes, haplotype diversity, variance of haplotype diversity and standard deviation of haplotype diversity.

Sequences obtained in this study were deposited in GenBank (Accession Numbers: MN650919-MN651079).

## 3. Results

### 3.1. Leporids IGHE and IGHM

We newly sequenced the *IGHM and IGHE* genes for six leporid genera: *Bunolagus*, *Brachylagus*, *Lepus*, *Pentalagus*, *Romerolagus* and *Sylvilagus*. The gene structure is similar to the European rabbit *IGHM and IGHE* and we thus inferred the intron-exon organization from published rabbit *IGHM and IGHE* sequences. Splicing signals are present at the intron boundaries and therefore we assumed that all studied leporids share the same *IGHM and IGHE* exon organization as the European rabbit.

#### 3.1.1. IGHM

##### Cµ1 Domain

**Figure 3:**
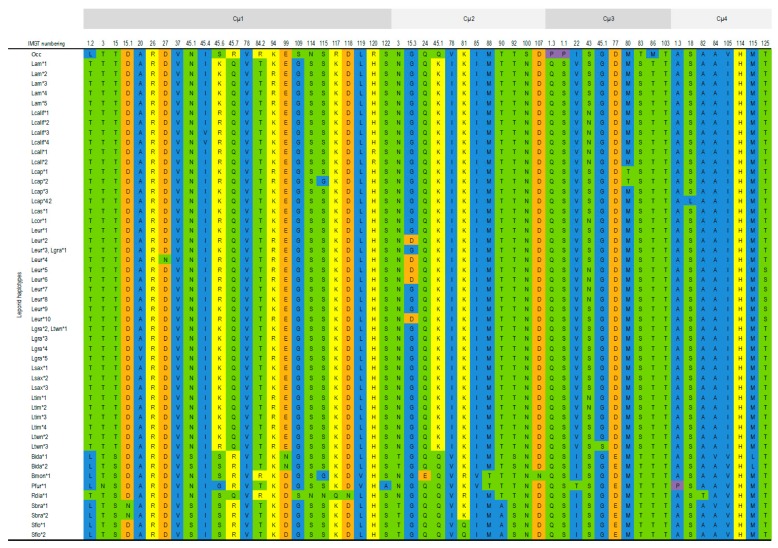
Variable codons and amino acid physicochemical properties for leporid *IGHM* constant domains. The codon numbering is according to the IMGT unique numbering for the constant domain (13). The colours represent amino acid properties: polar neutral (green), polar positive (yellow), polar negative (orange), non-polar neutral (purple), non-polar aliphatic (blue) and non-polar aromatic (light pink). Occ—*Oryctolagus cuniculus cuniculus* (accession number J00666); Lcalif—*Lepus californicus*; Lcal—*Lepus callotis*; Lcas—*Lepus castroviejoi*; Lcap—*Lepus* capensis; Leur—*Lepus europaeus*; Lgra—*Lepus granatensis*; Lsax—*Lepus saxatilis;* Ltwn—*Lepus townsendi*; Lam—*Lepus americanus;* Lcap—*Lepus capensis*; Ltim—*Lepus timidus*; Pfur—*Pentalagus furnessii*; Sflo—*Sylvilagus floridanus*; Sbac—*Sylvilagus bachmanii*; Rdia—*Romerolagus diazii*; Bida—*Brachylagus idahoensis*; Bmon—*Bunolagus monticularis.*

The Cµ1 domain is the most varied of leporid *IGHM* domains with 24 amino acid variable positions, the majority of which involve one substitution and 11 are conservative regarding the amino acid properties. For the Cµ1 domain some genera specific residues are observed: Arg 117 for the European rabbit, Asn 3 and Ala 122 for *Pentalagus* and Asn 115, Gln 117 and Asn 118 for *Romerolagus* (Figure 3).

##### Cµ2 Domain

The leporid Cµ2 domain is fairly conserved with only 12 amino acid variable positions. The great majority of these polymorphic positions involve one substitution but seven encode for changes in amino acid properties. The European rabbit has specific Ser residues at positions 100, while *Lepus* have specific Lys 45 residues and *Sylvilagus* have Ala 90 residues (Figure 3).

##### Cµ3 Domain

The variability observed for the leporid Cµ3 domain is similar to that of the Cµ2 domain. Of the 10 variable positions present in the CH3 domain all but one involve one substitution but seven encode for changes in amino acid properties. Interestingly, the European rabbit has two Pro residues at positions 1.3 and 1.1 and Met 86 residues whereas other leporids have 1.3 GluSer 1.1 and Thr 86. L*epus* IgM specifically has Val 22 and Ser 83 (Figure 3).

##### Cµ4 domain

This is the most conserved of the leporid *IGHM* domains with 8 variable positions, half of which contain changes in amino acid properties. In this domain only one diagnostic position was observed for two genera: *Lepus* at Ile105 and *Brachylagus* at Leu 115 (Figure 3).

#### 3.1.2. IGHE

##### Cε1 Domain

**Figure 4:**
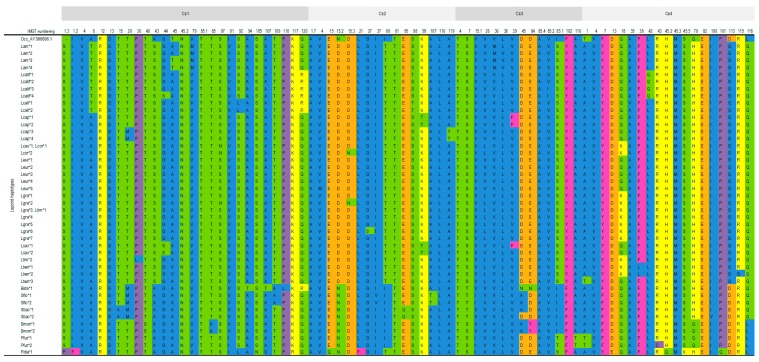
Variable codons and amino acid physicochemical properties for leporid *IGHE* constant domains. The codon numbering is according to the IMGT unique numbering for the constant domain (13). The colours represent amino acid properties: polar neutral (green), polar positive (yellow), polar negative (orange), non-polar neutral (purple), non-polar aliphatic (blue) and non-polar aromatic (light pink). Occ—*Oryctolagus cuniculus cuniculus* (accession number AY386696.1); Lcalif—*Lepus californicus*; Lcall—*Lepus callotis*; Lcas—*Lepus castroviejoi*; Lcap—*Lepus* capensis; Leur—*Lepus europaeus*; Lgra—*Lepus granatensis*; Lsax—*Lepus saxatilis;* Ltwn—*Lepus townsendi*; Lam—*Lepus americanus;* Lcap—*Lepus capensis*; Ltim—*Lepus timidus*; Pfur—*Pentalagus furnessii*; Sflo—*Sylvilagus floridanus*; Sbac—*Sylvilagus bachmanii*; Rdia—*Romerolagus diazii*; Bida—*Brachylagus idahoensis*; Bmon—*Bunolagus monticularis.*

Similarly, to what was observed for *IGHM,* the Cε1 domain is the most diverse of leporid *IGHE* domains. In this domain 27 amino acid variable positions were observed. Of these, only four involve more than one substitution and six encode changes in amino acid properties. Specific residues were observed for *Bunolagus*, Ala 13, *Lepus*, Ser 43, and *Romerolagus*, Pro 1.3 and Phe 1.2 (Figure 4).

##### Cε2 Domain

In the leporid Cε2 domain 16 amino acid variable positions were observed. These involve one substitution but the majority encodes for changes in amino acid properties. Two diagnostic positions were observed for *Romerolagus* Gln15 and Phe21 (Figure 4).

##### Cε3 Domain

The leporid Cε3 domain has 14 amino acid variable positions, two of which involve more than one substitution and five encode for changes in amino acid properties. Two genera specific positions were observed for each of three genera: *Lepus* has specific Glu84 and Tyr102, *Brachylagus* has characteristic Asn45.4 and Asn84 and *Pentalagus* has as distinctive residues Thr85.1 and Thr116 (Figure 4).

##### Cε4 Domain

Contrarily to that observed for leporid *IGHM,* the Cε4 domain has higher variability than the Cε2 and Cε3 domains. The Cε4 domain has 19 amino acid variable positions, 13 of which involve changes in amino acid properties. *Lepus* has specific Glu 113 residues, *Pentalagus* has specific Cys 43 residues and *Romerolagus* has specific Glu13, Gln 45.2, Lys 45.5, and Gln 101 residues (Figure 4). 

### 3.2. Wild and Domestic European Rabbits IgM and IgE Diversity

#### 3.2.1. IGHM

European rabbits *IGHM* diversity seems to be higher for the *O. c. cuniculus* subspecies: of the 31 SNP’s we found, 20 were observed for the Spanish *O. c. cuniculus* population and 15 for the French *O. c. cuniculus* population, while 13 and 11 were found for the *O. c. algirus* subspecies and domestic breeds, respectively (Table 1). The nucleotide diversity agrees with this pattern (Table 1). Forty haplotypes were recovered (Table 1) most of which are rare, occurring once in heterozygosity, or uncommon (0.01–0.10 frequencies). The most common haplotype (0.178 frequency) is shared between *O. c. algirus* and the domestic breeds. Other haplotypes are shared between *O. c. algirus* and French wild *O. c. cuniculus*, *O. c. cuniculus* and domestic breeds and between French wild *O. c. cuniculus* and domestic breeds. The haplotype diversity values are in accordance with the nucleotide diversity, being highest for the French wild *O. c. cuniculus* and lowest for the domestic breeds (Table 1).

Less than one quarter of the recovered SNPs involves nonsynonymous substitutions resulting in 7 polymorphic amino acid positions (Table 2). Unique amino acids were found for *O. c. algirus*, G at CH1 residue 95, and Spanish wild *O. c. cuniculus*, I at CH3 residue 103, T and L at CH4 residues 82 and 114, respectively (Table 2).

#### 3.2.2. IGHE

Our results show a greater diversity of IGHE in the IP populations: of the 26 SNPs observed, 16 were found for the *O. c. algirus* and 16 other for the Spanish wild *O. c. cuniculus* (Table 1). Surprisingly, for the Domestic breeds 9 SNPs were observed but only three SNPs were observed for the French *O. c. cuniculus* (Table 1). The nucleotide diversity values are in agreement with the observed SNPs (Table 1). We recovered 24 haplotypes, two of which are common: one haplotype is shared among all populations (0.322 frequency) and another haplotype is shared between *O. c. cuniculus* French populations and domestic breed’s (0.267 frequency); the remaining haplotypes are rare or uncommon (0.01–0.10 frequencies). 

Approximately half of the recovered SNP’s involve nonsynonymous substitutions resulting in 12 polymorphic amino acid positions (Table 3). Unique amino acids were found for *O. c. algirus*: M and F at CH2 residues 81 and 116, respectively; Spanish wild *O. c. cuniculus*: I and M at CH1 residues 4 and 84, respectively, and K at CH4 residue 82; and for the domestic breeds: K, A and T at CH1 residues 12, 44 and 45, respectively, G at CH2 residue 1.7 and V at CH3 residue 85 (Table 3)

## 4. Discussion

The study of European rabbit immunoglobulin genes has contributed to elucidating numerous fundamental aspects of antibody structure and diversification mechanisms (reviewed in [36]). The rabbit has also been increasingly used as an animal model to many human diseases [2]. Despite this, the study of rabbit immunoglobulins has focused on the IgG and IgA isotypes. The knowledge on other leporids immune system genes is also very scarce. 

### 4.1. IGHM and IGHE Evolution

The results obtained in this study show that throughout Leporid evolution *IGHM* and *IGHE* have been fairly conserved (each Ig has 98% sequence similarity among leporids), similarly to what has been found for *IGHG* [11]. A gradient in *IGHM* constant domains diversity was observed, with the Cµ1 domain being the most diverse and the Cµ4 the most conserved. This gradient in domain diversity was not observed for *IGHE*, the Cε1 is the most diverse domain but Cε4 shows greater diversity than Cε2 or Cε3 domains, the most conserved domains. For leporid *IGHG* a gradient in the constant domains diversity was described from the most conserved Cγ1 domain to the most diverse Cγ3 domain [11] and for the European rabbit IgA subclasses the Cα1 domain has twice more variable amino acid sites than Cα2 and Cα3 domains [6,7]. The differences in the pattern of most conserved and most diverse domain between the different Ig isotypes are most likely related to each isotype function. To trigger the effector functions that lead to pathogen elimination Igs must bind to other effector molecules such as Fc receptors and the complement. IgM interacts with three receptors, FcµR, Fcα/µR and pIgR, the binding sites of which have been mapped to the Cµ4 domain [37,38,39] and with the complement which binds to sites in the Cµ3 domain [40,41,42], the domains we found to be the most conserved during leporid IGHM evolution. IgE two receptors, FcεRI and FcεRII, binding sites locate to the Cε3 [43,44], again the domain we found to be the most conserved. IgA ligands, both Fc receptors, FcαRI and pIgR, and some pathogen evasion proteins interact with sites on the Cα2 and Cα3 domains (reviewed in [45], the most conserved IgA domains in rabbit IgA subclasses [6,7] and during mammalian evolution [45]. As for rabbit IgG, it’s receptor, FcγR, and the complement C1q protein have been shown to bind residues in the Cγ2 domain [46,47,48] a very conserved domain in leporid IgG evolution [11]. Thus, Ig domains which are likely essential for interaction with host ligands have been conserved during leporid evolution. Curiously, for the IgE Cε3 domain all but two of the 14 amino acid variable positions we found, including the five positions that encode for changes in amino acid properties, lie in or in direct vicinity of the binding sites to FcεRI and FcεRII. Variation at these codons may prove adaptive by improving the binding of IgE to its Fc receptors. It would be interesting to study the rabbit IgE Fc receptors which may also show specific differences reflecting the host adaptation to different selective pressures imposed by the different environments that each leporid species inhabit. 

### 4.2. Wild and Domestic European Rabbits IgM and IgE Diversity

The IP is the region were the European rabbit originated [13]. Later, during the 7^th^ century the rabbit was domesticated from French wild European rabbit populations [15,16]. Consistent with this, immunological markers genetic diversity has been shown to decrease from the IP to the French wild European rabbit populations and that the domestic animals are, to a great extent, a subset of the diversity found in the French wild *O. c. cuniculus* [29,30,36,49,50,51,52]. The results we obtained for IgE show a greater nucleotide and haplotype diversity in the IP *O.c. algirus* populations, in agreement with the previously described pattern of highest genetic diversity in IP populations. Interestingly, the genetic diversity we found for the domestic animals IgE is higher than that for the French wild *O. c. cuniculus* (domestic animals nucleotide diversity is twice as high as for French *O. c. cuniculus* and haplotype diversity is 1,5 times higher for domestic animals, Table 1), similar to what was found for European rabbit IgG [29]. This may reflect changes in allele frequencies caused by the founder effect on the origin of domestic breeds [15]. Striking for IgE is also the fact that despite the highest number of SNPs was found for the wild Iberian populations, the higher number of unique amino acids was found for the domestic animals, again similarly to what was found for IgG [29]. This indicates that the domestic breeds are under a higher selective pressure, possibly due to higher densities leading to exposure to different parasitic worms, leading to the emergence and maintenance of mutations of functional significance. In fact, despite the prophylaxis adopted in rabbitries, the prevalence of parasitic infections in domestic rabbits is high and mixed infestations predominate (approx. 80%; [53,54]) as an excessive stocking density of animals favors invasion mechanisms [54]. On the other hand, the more ancient Iberian European rabbit populations have accumulated a higher number of random mutations. We cannot discard that the higher variability in the domestic rabbits could be associated with a compensatory mechanism already proposed by van der Loo [55]: to compensate the lack of genetic diversity shown by the rabbit domestic breeds for most immunologic genes, some particular genes show a higher diversity when compared to the wild populations.

The results we obtained for IgM, on the other hand, revealed a higher nucleotide and haplotype diversity for the *O. c. cuniculus* subspecies, particularly for the French populations, and that the domestic animals have the lowest genetic diversity (Table 1). Considering the results previously obtained for most immunological markers [29,30,36,49,50,51,52] the higher genetic diversity observed for the French *O. c. cuniculus* was unexpected. Nevertheless, this higher nucleotide and haplotype diversity does not translate into higher number of polymorphic amino acid sites for the French *O. c. cuniculus*. Noteworthy for IgM is also the fact that only a small proportion of the observed SNP’s (7 out of 31 SNPs) involve non-synonymous substitutions, in spite of the observed 31 SNPs only seven polymorphic amino acid positions were detected. IgM is the most ancient Ig isotype being present with the same function in all gnathostomes [56]. As such it is a very conserved gene that most likely suffers stochastic nucleotide substitutions and is subject to strong purifying selection.

## 5. Conclusions

Our findings show that throughout Leporid evolution *IGHM* and *IGHE* have been fairly conserved. Despite this, variable amino acid residues were identified for IgE which may improve the binding of IgE to its Fc receptors. The observed pattern of genetic diversity of *IGHM* and *IGHE* along the wild rabbit populations from Iberia and France is contradictory, being, as expected, higher for the Iberian populations for *IGHE* but surprisingly higher for French populations for *IGHM*. This is the first time that these two immunoglobulin isotypes were fully characterized for Leporids and for European rabbit wild populations and domestic breeds. These findings will be useful for the management of rabbit populations and for the understanding of the rabbit adaptive immunity against pathogens.

## Figures and Tables

**Table 1:** *IGHM* and *IGHE* nucleotide sequence polymorphism and estimates of haplotype diversity, haplotype diversity variance and haplotype standard deviation in the European rabbit populations/domestic animals studied.

Locus	Subspecies	Population	N	S	*π*	D	H	Hd	Hd Variance	Hd Std Deviation
IGHM		All	45	31	0.00317	−0.98878	40	0.949	0.00017	0.013
	*O. c. algirus*	Portugal	10	13	0.00283	0.10630	11	0.911	0.00176	0.042
	*O. c. cuniculus*	Spain	10	20	0.00340	−0.84715	13	0.911	0.00289	0.054
		France	10	15	0.00345	0.39814	13	0.958	0.00065	0.025
		Domestic	15	11	0.00243	0.59465	8	0.747	0.00363	0.060
IGHE		All	45	26	0.00226	−1.38819	24	0.817	0.00078	0.028
	*O. c. algirus*	Portugal	10	16	0.00348	−0.02986	11	0.868	0.0041	0.064
	*O. c. cuniculus*	Spain	10	16	0.00222	−1.37449	8	0.774	0.0051	0.071
		France	10	3	0.00068	0.08868	3	0.542	0.01094	0.105
		Domestic	15	9	0.00136	−0.71769	8	0.743	0.003	0.055

N, sample size; S, segregating sites; *π*, nucleotide diversity; D, Tajima’s D statistical test; H, number of haplotypes; Hd, haplotype diversity; Hd variance, variance of haplotype diversity; Hd std deviation, standard deviation of haplotype diversity.

**Table 2:** IgM amino acid polymorphic positions for European rabbit populations/domestic animals. The nucleotide variation is shown, dots represent identity. The codon numbering is according to the IMGT unique numbering for the constant domain [33].

**Subspecies**	**Population**	**Domain, Amino Acid Position and Most Common Triplet and Amino Acid**													
		Cµ1								Cµ3		Cµ4			
		26		37		78		109		103		82		114	
		cgg	R	gtc	V	gtc	V	agc	S	acg	T	gcc	A	cac	H
*O. c. algirus*	Portugal	…	R	…	V	…	V	ga.	D	…/..a	T	…	A	…	H
		.a.	Q	a..	I			…	S						
								g..	G						
*O. c. cuniculus*	Spain	…	R	…	V	…	V	ga.	D	…/..a	T	…	A	…	H
		.a.	Q	a..	I			…	S	.ta	I	a..	T	.t.	L
	France	…	R	…	V	…	V	ga.	D	…/..a	T	…	A	…	H
				a..	I	a..	I	…	S						
	Domestic	…	R	…	V	…	V	ga.	D	…/..a	T	…	A		
				a..	I	a..	I	…	S						

**Table 3:** IgE amino acid polymorphic positions for European rabbit populations/domestic animals. The nucleotide variation is shown, dots represent identity. The codon numbering is according to the IMGT unique numbering for the constant domain [33].

Subspecies	Population	Domain, Amino Acid Position and Most Common Triplet and Amino Acid																							
		Cε1										Cε2						Cε3				Cε4			
		4		12		44		45.2		84.4		1.7		81		116		84		85.4		1		82	
		gtc	V	aga	R	ggc	G	aac	N	acg	T	gcg	A	acg	T	ctc	L	tat	Y	gcc	A	acc	T	gag	E
*O. c. algirus*	Portugal	…	V	…	R	…	G	…	N	…	T	…	A	…	T		L	…	Y	…	A	…	T	…	E
														.t.	M	t..	F	g..	D			g..	A		
*O. c. cuniculus*	Spain	…	V	…	R	…	G	…	N	…	T	…	A	…	T	…	L	g..	D	…	A	….	T	…	E
		a..	I							.t.	M							…	Y			g..	A	a..	K
	France	…	V	…	R	…	G	…	N	…	T	…	A	…	T	…	L	g..	D	…	A	…	T	…	E
																						g..	A		
	Domestic	…	V	…	R	…	G	…	N	…	T	…	A	…	T	…	L	g..	D		A	…	T	…	E
				.a.	K	.c.	A	.c.	T			.g.	G							.t.	V	g..	A		
